# *N*-(4-Meth­oxy­benz­yl)phthalimide: a triclinic polymorph

**DOI:** 10.1107/S1600536812031376

**Published:** 2012-07-14

**Authors:** Hiroki Takahashi

**Affiliations:** a Graduate School of Human and Environmental Studies, Kyoto University, Kyoto 606-8501, Japan

## Abstract

The title compound [systematic name: 2-(4-meth­oxy­benz­yl)­isoindoline-1,3-dione], C_16_H_13_NO_3_, represents a triclinic polymorph of the previously reported monoclinic form [Warzecha *et al.* (2006 ▶). *Acta Cryst*. E**62**, o5450–o5452]. The reaction of potassium phthalimide and 4-meth­oxy­benzyl chloride in dimethyl­formamide gave platelet-shaped crystals; these were harvested and then needle-shaped crystals were deposited. The platelet- and needle-shaped crystals correspond to the triclinic and monoclinic forms, respectively. The N—C—C_ar_—C_ar_ torsion angles between the ring systems are −82.66 (14) and 95.28 (13)°, resulting in a roof-shaped conformation. In the crystal, mol­ecules are accumulated by offset face–face π–π inter­actions between phthalimide units [centroid–centroid distances = 3.640 (2) and 3.651 (2) Å], with inter­planar distances of 3.321 (1) and 3.435 (1) Å. Weak inter­molecular C_ar­yl_—H⋯O=C and C_alk­yl_—H⋯O=C contacts form *C*(8) and *C*(11) infinite chain motifs, respectively.

## Related literature

For the crystal structure of the monoclinic form of the title compound, see: Warzecha *et al.* (2006*b* ▶). For a photochemical study of the title compound, see: Warzecha, Görner *et al.* (2006 ▶). For related compounds, see: Lü *et al.* (2006 ▶); Warzecha *et al.* (2006*a* ▶); Chen *et al.* (2006 ▶). For graph-set motifs, see: Etter (1990 ▶).
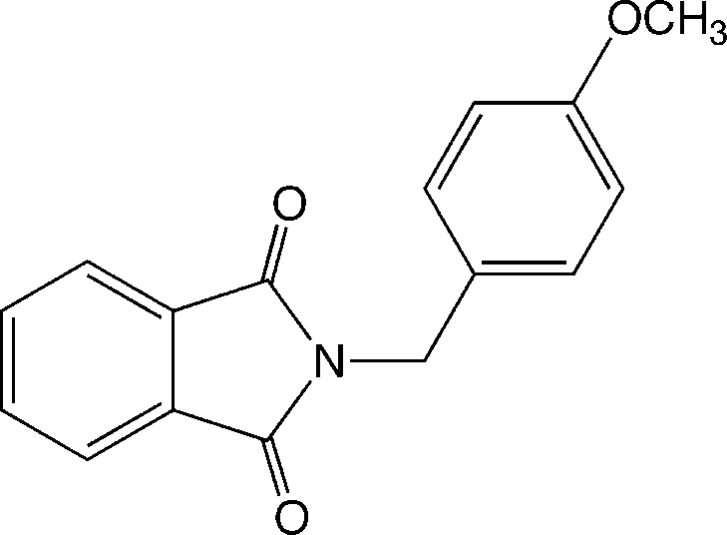


## Experimental

### 

#### Crystal data

C_16_H_13_NO_3_
*M* *_r_* = 267.27
Triclinic, 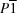

*a* = 8.190 (3) Å
*b* = 8.293 (4) Å
*c* = 11.465 (5) Å
α = 105.794 (5)°
β = 90.8094 (16)°
γ = 118.154 (5)°
*V* = 651.3 (5) Å^3^
*Z* = 2
Mo *K*α radiation
μ = 0.10 mm^−1^
*T* = 100 K
0.35 × 0.30 × 0.05 mm

#### Data collection

Rigaku Saturn724+ diffractometer
Absorption correction: multi-scan (*ABSCOR*; Higashi, 1995 ▶) *T*_min_ = 0.967, *T*_max_ = 0.995
6640 measured reflections
2951 independent reflections
2256 reflections with *I* > 2σ(*I*)
*R*_int_ = 0.019

#### Refinement

*R*[*F* ^2^ > 2σ(*F* ^2^)] = 0.034
*wR*(*F* ^2^) = 0.087
*S* = 0.96
2951 reflections
212 parameters
H atoms treated by a mixture of independent and constrained refinement
Δρ_max_ = 0.26 e Å^−3^
Δρ_min_ = −0.17 e Å^−3^

### 

Data collection: *CrystalClear-SM Expert* (Rigaku/MSC, 2009 ▶); cell refinement: *CrystalClear-SM Expert*; data reduction: *CrystalClear-SM Expert*; program(s) used to solve structure: *SIR97* (Altomare *et al.*, 1999 ▶); program(s) used to refine structure: *SHELXL97* (Sheldrick, 2008 ▶); molecular graphics: *Yadokari-XG 2009* (Kabuto *et al.*, 2009 ▶), *ORTEP-3* (Farrugia, 1997 ▶) and *Mercury* (Macrae *et al.*, 2008 ▶); software used to prepare material for publication: *PLATON* (Spek, 2009 ▶) and *publCIF* (Westrip, 2010 ▶).

## Supplementary Material

Crystal structure: contains datablock(s) I, global. DOI: 10.1107/S1600536812031376/bh2442sup1.cif

Structure factors: contains datablock(s) I. DOI: 10.1107/S1600536812031376/bh2442Isup2.hkl

Supplementary material file. DOI: 10.1107/S1600536812031376/bh2442Isup3.cml

Additional supplementary materials:  crystallographic information; 3D view; checkCIF report

## Figures and Tables

**Table 1:** Hydrogen-bond geometry (Å, °)

*D*—H⋯*A*	*D*—H	H⋯*A*	*D*⋯*A*	*D*—H⋯*A*
C5—H3⋯O1^i^	0.959 (14)	2.422 (14)	3.245 (2)	143.7 (10)
C16—H13⋯O2^ii^	0.98	2.53	3.287 (2)	134

Symmetry codes: (i) 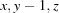; (ii) 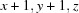.

**Fig. 1.**
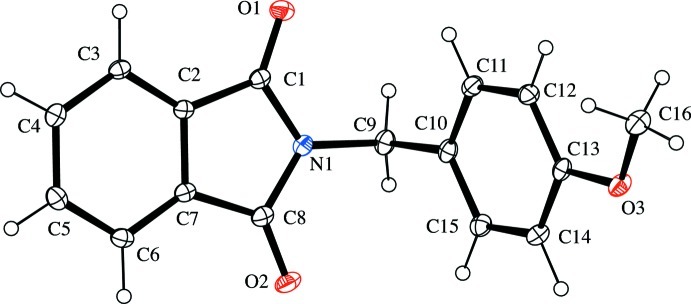
The molecular structure of the title compound, showing displacement ellipsoids at the 50% probability level; H atoms are shown as circles of arbitrary size.

**Fig. 2.**
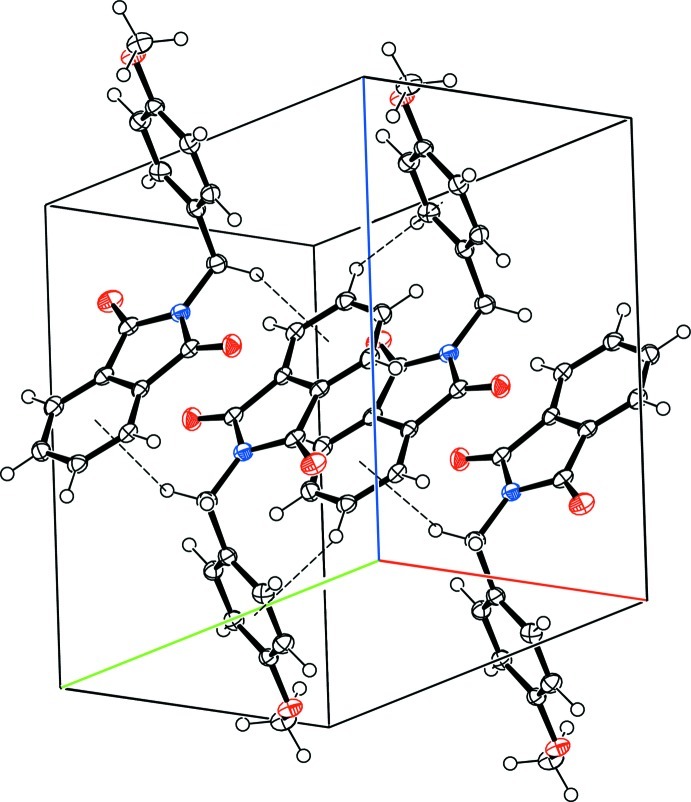
A view of the unit-cell content of the title compound, showing offset face-face π-π stacking and CH···π interactions. Dashed lines indicate CH···π interactions.

**Fig. 3.**
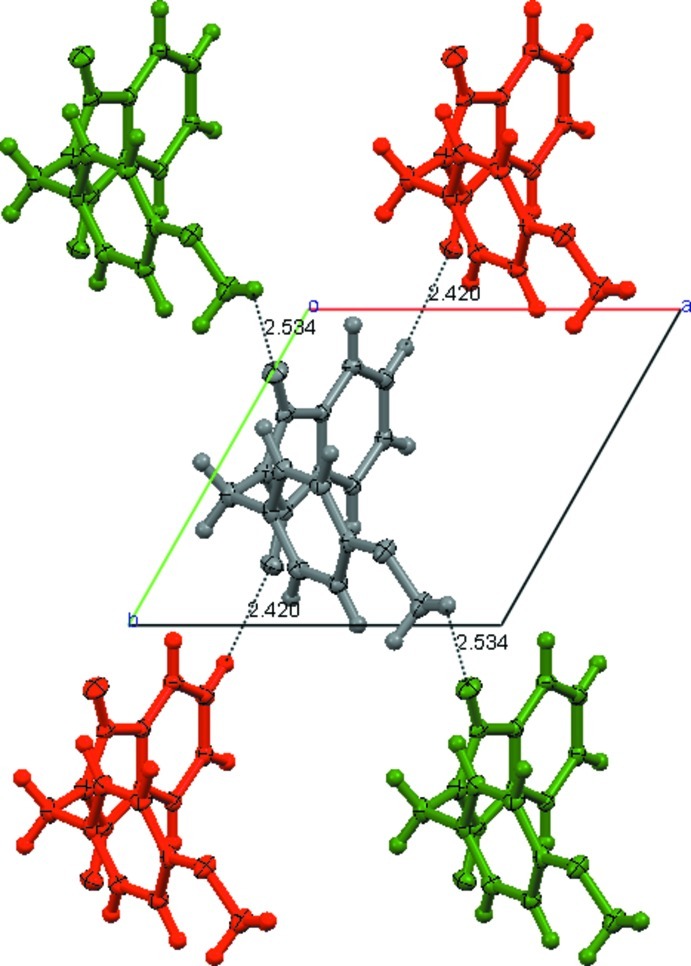
A view of the unit-cell contents of the title compound; red-gray-red molecules and green-gray-green molecules show C(8) and C(11) motifs, respectively. Dotted lines indicate C—H···O=C hydrogen bonds.

## supplementary crystallographic information

### Comment

Warzecha, *et al.* (2006*b*) reported the crystal structure of the monoclinic form of title compound, which was obtained from an ethanolic solution. Herein, the crystal structure of a triclinic form obtained from a dimethylformamide solution is described. The molecule (Fig. 1), consists of two planar subunits, *i.e*. a phthalimide moiety and a benzene ring, being linked by a *sp*^3^-C9 atom so that the molecular conformation is characterized by the N1—C9—C10—C11 and N1—C9—C10—C15 torsion angles. Torsion angle corresponding to N1—C9—C10—C11 in the monoclinic form is 153.41 (14)° whereas that of the triclinic form is -82.66 (14)°. The torsion angle in the triclinic form is almost the same as the triclinic form of *N*-benzylphthalimide [86.9 (3)°, Lü *et al.*, 2006 and -86.82 (16)°, Warzecha *et al.*, 2006*a*] and *N*-(4-methlybenzyl)phthalimide [84.2 (3)°, Chen *et al.*, 2006].

In the crystal structure of the triclinic form (Fig. 2), molecules are arranged *via* offset face-face π-π interactions between phthalimides in a head-to-tail fashion [interplanar distances (-*x*, 1 - *y*, 1 - *z*) = 3.321 (1) Å and (1 - *x*, 1 - *y*, 1 - *z*) = 3.435 (1) Å]. C—H···π interactions are observed between C4—H2 and the centroid of the benzene ring [(1 - *x*, 1 - *y*, 1 - *z*) = 2.818 (13) Å], and C9—H6 and the centroid of the six-membered ring of the phthalimide [(-*x*, 1 - *y*, 1 - *z*) = 2.996 (15) Å]. In the monoclinic crystal, C_aryl_—H···O=C and C_aryl_—H···O—C_aryl_ hydrogen bonds form *R*_2_^2^(16) and *R*_4_^4^(22) motifs (Etter, 1990), respectively, while in the triclinic form, C_aryl_—H···O=C and C_alkyl_—H···O=C interactions form *C*(8) and *C*(11) infinite chain structures (Fig. 3 and Table 1), respectively.

### Experimental

Reagents for the synthesis were from Tokyo Chemical Industry Co. and they were used without additional purification. A mixture of potassium phthalimide (0.926 g, 5 mmol) and 4-methoxybenzyl chloride (0.80 g, 5.1 mmol) in dimethylformamide (5 ml) was stirred at ambient temperature. The resulting suspension became a clear solution after 2 h. The solution was allowed to stand for a further 12 h without stirring. Several colourless platelet single crystals were deposited and used for X-ray analysis and then the crystal shape changed from platelet into needle for another 12 h. The resulting needle crystal was isolated by filtration, and successively washed with cold water and EtOH. Recrystallization from EtOH yielded colourless needle crystals (1.07 g, 80%, m.p. 403 K). The melting point of the platelet crystal was also 403 K. The cell parameters of a needle crystal corresponded to the monoclinic form.

### Refinement

The H atoms of the methoxy group were positioned with idealized geometry with C—H = 0.98 Å and refined as riding with *U_iso_*(H) = 1.5*U*_eq_(C16). All other H atoms were located in a difference Fourier map and refined freely with *U*_iso_(H) = 1.2*U*_eq_(C).

### Crystal data

**Table:**

C_16_H_13_NO_3_	*Z* = 2
*M**_r_* = 267.27	*F*(000) = 280
Triclinic, *P*1	*D*_x_ = 1.363 Mg m^−^^3^
Hall symbol: -P 1	Melting point: 403 K
*a* = 8.190 (3) Å	Mo *K*α radiation, λ = 0.71075 Å
*b* = 8.293 (4) Å	Cell parameters from 2075 reflections
*c* = 11.465 (5) Å	θ = 3.1–27.5°
α = 105.794 (5)°	µ = 0.10 mm^−^^1^
β = 90.8094 (16)°	*T* = 100 K
γ = 118.154 (5)°	Platelet, colourless
*V* = 651.3 (5) Å^3^	0.35 × 0.30 × 0.05 mm

### Data collection

**Table:**

Rigaku Saturn724+ diffractometer	2951 independent reflections
Radiation source: fine-focus sealed tube	2256 reflections with *I* > 2σ(*I*)
Graphite Monochromator monochromator	*R*_int_ = 0.019
Detector resolution: 28.5714 pixels mm^-1^	θ_max_ = 27.5°, θ_min_ = 3.2°
profile data from ω–scans	*h* = −10→10
Absorption correction: multi-scan (*ABSCOR*; Higashi, 1995)	*k* = −10→9
*T*_min_ = 0.967, *T*_max_ = 0.995	*l* = −14→14
6640 measured reflections	

### Refinement

**Table:**

Refinement on *F*^2^	Primary atom site location: structure-invariant direct methods
Least-squares matrix: full	Secondary atom site location: difference Fourier map
*R*[*F*^2^ > 2σ(*F*^2^)] = 0.034	Hydrogen site location: inferred from neighbouring sites
*wR*(*F*^2^) = 0.087	H atoms treated by a mixture of independent and constrained refinement
*S* = 0.96	*w* = 1/[σ^2^(*F*_o_^2^) + (0.0536*P*)^2^] where *P* = (*F*_o_^2^ + 2*F*_c_^2^)/3
2951 reflections	(Δ/σ)_max_ = 0.001
212 parameters	Δρ_max_ = 0.26 e Å^−^^3^
0 restraints	Δρ_min_ = −0.17 e Å^−^^3^
0 constraints	

### Fractional atomic coordinates and isotropic or equivalent isotropic displacement parameters (Å^2^)

**Table:**

	*x*	*y*	*z*	*U*_iso_*/*U*_eq_	
C1	0.24334 (15)	0.63806 (15)	0.48690 (9)	0.0191 (2)	
C2	0.28334 (14)	0.51357 (14)	0.54106 (9)	0.0171 (2)	
C3	0.38757 (15)	0.55798 (16)	0.65211 (9)	0.0191 (2)	
H1	0.4508 (16)	0.6886 (17)	0.7095 (11)	0.023*	
C4	0.40053 (15)	0.40875 (16)	0.67783 (10)	0.0214 (2)	
H2	0.4719 (16)	0.4320 (16)	0.7563 (11)	0.026*	
C5	0.31087 (16)	0.22283 (16)	0.59444 (10)	0.0233 (3)	
H3	0.3180 (17)	0.1217 (18)	0.6154 (11)	0.028*	
C6	0.20666 (16)	0.17978 (16)	0.48220 (10)	0.0218 (2)	
H4	0.1445 (17)	0.0499 (17)	0.4223 (11)	0.026*	
C7	0.19461 (14)	0.32803 (15)	0.45735 (9)	0.0185 (2)	
C8	0.09817 (15)	0.32978 (15)	0.34721 (9)	0.0204 (2)	
C9	0.07198 (16)	0.58653 (18)	0.28326 (10)	0.0234 (2)	
H5	−0.0527 (18)	0.4833 (17)	0.2403 (11)	0.028*	
H6	0.0580 (17)	0.6973 (18)	0.3337 (12)	0.028*	
C10	0.20838 (15)	0.64133 (16)	0.19466 (9)	0.0206 (2)	
C11	0.36928 (16)	0.82062 (16)	0.22606 (10)	0.0222 (2)	
H7	0.3937 (17)	0.9133 (17)	0.3059 (11)	0.027*	
C12	0.49800 (16)	0.86831 (16)	0.14608 (10)	0.0216 (2)	
H8	0.6131 (17)	0.9974 (17)	0.1698 (11)	0.026*	
C13	0.46131 (15)	0.73395 (16)	0.03122 (9)	0.0198 (2)	
C14	0.30067 (16)	0.55359 (16)	−0.00133 (10)	0.0229 (2)	
H9	0.2783 (16)	0.4566 (17)	−0.0834 (11)	0.027*	
C15	0.17604 (16)	0.50759 (17)	0.08016 (10)	0.0227 (2)	
H10	0.0627 (17)	0.3755 (17)	0.0568 (11)	0.027*	
C16	0.75343 (15)	0.93637 (17)	−0.02176 (10)	0.0277 (3)	
H11	0.7368	1.0498	−0.0019	0.042*	
H12	0.8246	0.9363	−0.0897	0.042*	
H13	0.8222	0.9402	0.0505	0.042*	
O1	0.29209 (11)	0.80774 (11)	0.52998 (7)	0.0264 (2)	
O2	0.00784 (11)	0.19931 (11)	0.25296 (7)	0.0285 (2)	
N1	0.13193 (13)	0.51842 (13)	0.37221 (8)	0.0202 (2)	
O3	0.57369 (10)	0.76553 (11)	−0.05703 (7)	0.0243 (2)	

### Atomic displacement parameters (Å^2^)

**Table:**

	*U*^11^	*U*^22^	*U*^33^	*U*^12^	*U*^13^	*U*^23^
C1	0.0172 (5)	0.0215 (6)	0.0175 (5)	0.0096 (5)	0.0056 (4)	0.0045 (4)
C2	0.0151 (5)	0.0175 (5)	0.0180 (5)	0.0078 (4)	0.0063 (4)	0.0048 (4)
C3	0.0169 (5)	0.0202 (6)	0.0178 (5)	0.0088 (5)	0.0042 (4)	0.0030 (4)
C4	0.0186 (5)	0.0276 (6)	0.0198 (5)	0.0123 (5)	0.0056 (4)	0.0084 (5)
C5	0.0238 (6)	0.0232 (6)	0.0282 (6)	0.0137 (5)	0.0094 (5)	0.0120 (5)
C6	0.0211 (6)	0.0172 (5)	0.0236 (6)	0.0076 (5)	0.0067 (4)	0.0044 (4)
C7	0.0148 (5)	0.0193 (5)	0.0175 (5)	0.0065 (4)	0.0049 (4)	0.0040 (4)
C8	0.0169 (5)	0.0221 (6)	0.0184 (5)	0.0076 (5)	0.0067 (4)	0.0045 (4)
C9	0.0221 (6)	0.0316 (6)	0.0214 (5)	0.0159 (5)	0.0047 (5)	0.0106 (5)
C10	0.0203 (5)	0.0285 (6)	0.0184 (5)	0.0154 (5)	0.0025 (4)	0.0090 (4)
C11	0.0263 (6)	0.0250 (6)	0.0169 (5)	0.0152 (5)	0.0019 (4)	0.0041 (4)
C12	0.0213 (6)	0.0208 (6)	0.0204 (5)	0.0096 (5)	0.0003 (4)	0.0050 (4)
C13	0.0193 (5)	0.0267 (6)	0.0165 (5)	0.0134 (5)	0.0025 (4)	0.0078 (4)
C14	0.0222 (6)	0.0250 (6)	0.0175 (5)	0.0108 (5)	0.0003 (4)	0.0027 (4)
C15	0.0191 (6)	0.0249 (6)	0.0209 (5)	0.0089 (5)	0.0002 (4)	0.0066 (5)
C16	0.0189 (6)	0.0330 (7)	0.0234 (6)	0.0078 (5)	0.0035 (4)	0.0071 (5)
O1	0.0311 (5)	0.0211 (4)	0.0262 (4)	0.0144 (4)	0.0032 (3)	0.0038 (3)
O2	0.0278 (4)	0.0271 (4)	0.0186 (4)	0.0086 (4)	−0.0012 (3)	−0.0008 (3)
N1	0.0211 (5)	0.0227 (5)	0.0171 (4)	0.0111 (4)	0.0040 (4)	0.0058 (4)
O3	0.0196 (4)	0.0284 (4)	0.0173 (4)	0.0073 (3)	0.0034 (3)	0.0049 (3)

### Geometric parameters (Å, º)

**Table:**

C1—O1	1.2122 (14)	C9—H5	0.974 (13)
C1—N1	1.3956 (14)	C9—H6	1.001 (13)
C1—C2	1.4903 (16)	C10—C11	1.3867 (16)
C2—C3	1.3795 (15)	C10—C15	1.3942 (15)
C2—C7	1.3921 (15)	C11—C12	1.3928 (16)
C3—C4	1.3969 (16)	C11—H7	0.967 (12)
C3—H1	0.974 (12)	C12—C13	1.3929 (15)
C4—C5	1.3928 (16)	C12—H8	0.995 (12)
C4—H2	0.987 (12)	C13—O3	1.3720 (13)
C5—C6	1.3940 (17)	C13—C14	1.3902 (16)
C5—H3	0.959 (13)	C14—C15	1.3834 (15)
C6—C7	1.3808 (16)	C14—H9	1.005 (12)
C6—H4	0.980 (12)	C15—H10	1.004 (12)
C7—C8	1.4871 (16)	C16—O3	1.4286 (14)
C8—O2	1.2129 (13)	C16—H11	0.9800
C8—N1	1.3970 (16)	C16—H12	0.9800
C9—N1	1.4701 (14)	C16—H13	0.9800
C9—C10	1.5135 (15)		
			
O1—C1—N1	124.83 (10)	H5—C9—H6	107.4 (10)
O1—C1—C2	129.26 (10)	C11—C10—C15	118.74 (10)
N1—C1—C2	105.90 (9)	C11—C10—C9	121.47 (10)
C3—C2—C7	121.69 (10)	C15—C10—C9	119.76 (11)
C3—C2—C1	130.35 (10)	C10—C11—C12	121.38 (10)
C7—C2—C1	107.96 (10)	C10—C11—H7	119.2 (7)
C2—C3—C4	117.31 (10)	C12—C11—H7	119.4 (7)
C2—C3—H1	121.1 (7)	C11—C12—C13	118.97 (11)
C4—C3—H1	121.5 (7)	C11—C12—H8	120.6 (7)
C5—C4—C3	121.03 (11)	C13—C12—H8	120.4 (7)
C5—C4—H2	118.2 (7)	O3—C13—C14	115.20 (9)
C3—C4—H2	120.8 (7)	O3—C13—C12	124.59 (10)
C4—C5—C6	121.18 (11)	C14—C13—C12	120.21 (10)
C4—C5—H3	119.5 (7)	C15—C14—C13	119.99 (10)
C6—C5—H3	119.3 (8)	C15—C14—H9	120.7 (7)
C7—C6—C5	117.47 (10)	C13—C14—H9	119.3 (7)
C7—C6—H4	120.6 (7)	C14—C15—C10	120.69 (11)
C5—C6—H4	121.9 (7)	C14—C15—H10	119.3 (7)
C6—C7—C2	121.32 (11)	C10—C15—H10	120.0 (7)
C6—C7—C8	130.48 (10)	O3—C16—H11	109.5
C2—C7—C8	108.19 (10)	O3—C16—H12	109.5
O2—C8—N1	124.84 (11)	H11—C16—H12	109.5
O2—C8—C7	129.29 (11)	O3—C16—H13	109.5
N1—C8—C7	105.88 (9)	H11—C16—H13	109.5
N1—C9—C10	112.27 (9)	H12—C16—H13	109.5
N1—C9—H5	106.8 (7)	C1—N1—C8	112.06 (9)
C10—C9—H5	111.5 (7)	C1—N1—C9	123.97 (10)
N1—C9—H6	105.7 (7)	C8—N1—C9	123.73 (9)
C10—C9—H6	112.7 (7)	C13—O3—C16	117.23 (8)
			
O1—C1—C2—C3	−1.23 (19)	C9—C10—C11—C12	177.70 (10)
N1—C1—C2—C3	179.51 (10)	C10—C11—C12—C13	1.45 (17)
O1—C1—C2—C7	179.10 (10)	C11—C12—C13—O3	177.89 (9)
N1—C1—C2—C7	−0.17 (11)	C11—C12—C13—C14	−1.53 (16)
C7—C2—C3—C4	−0.16 (15)	O3—C13—C14—C15	−179.04 (10)
C1—C2—C3—C4	−179.80 (10)	C12—C13—C14—C15	0.44 (17)
C2—C3—C4—C5	−0.22 (15)	C13—C14—C15—C10	0.78 (17)
C3—C4—C5—C6	0.56 (17)	C11—C10—C15—C14	−0.87 (16)
C4—C5—C6—C7	−0.48 (16)	C9—C10—C15—C14	−178.86 (10)
C5—C6—C7—C2	0.09 (16)	O1—C1—N1—C8	−179.86 (10)
C5—C6—C7—C8	179.06 (10)	C2—C1—N1—C8	−0.56 (11)
C3—C2—C7—C6	0.23 (16)	O1—C1—N1—C9	5.55 (16)
C1—C2—C7—C6	179.94 (9)	C2—C1—N1—C9	−175.15 (9)
C3—C2—C7—C8	−178.94 (9)	O2—C8—N1—C1	−178.80 (10)
C1—C2—C7—C8	0.77 (11)	C7—C8—N1—C1	1.01 (11)
C6—C7—C8—O2	−0.35 (19)	O2—C8—N1—C9	−4.19 (16)
C2—C7—C8—O2	178.72 (10)	C7—C8—N1—C9	175.62 (9)
C6—C7—C8—N1	179.84 (10)	C10—C9—N1—C1	91.16 (12)
C2—C7—C8—N1	−1.09 (11)	C10—C9—N1—C8	−82.81 (13)
N1—C9—C10—C11	−82.66 (14)	C14—C13—O3—C16	−170.86 (10)
N1—C9—C10—C15	95.28 (13)	C12—C13—O3—C16	9.69 (15)
C15—C10—C11—C12	−0.26 (16)		

### Hydrogen-bond geometry (Å, º)

**Table:**

*D*—H···*A*	*D*—H	H···*A*	*D*···*A*	*D*—H···*A*
C5—H3···O1^i^	0.959 (14)	2.422 (14)	3.245 (2)	143.7 (10)
C16—H13···O2^ii^	0.98	2.53	3.287 (2)	134

Symmetry codes: (i) *x*, *y*−1, *z*; (ii) *x*+1, *y*+1, *z*.
